# Tau deficiency inhibits classically activated macrophage polarization and protects against collagen-induced arthritis in mice

**DOI:** 10.1186/s13075-023-03133-4

**Published:** 2023-08-09

**Authors:** Meng Chen, Wenyu Fu, Huiyun Xu, Chuan-ju Liu

**Affiliations:** 1 Department of Orthopaedic Surgery, New York University Grossman School of Medicine, New York, NY USA; 2 School of Life Sciences, Northwestern Polytechnical University, Xi’an, China; 3 Department of Orthopaedics and Rehabilitation, Yale University School of Medicine, New Haven, CT USA; 4 Department of Cell Biology, New York University Grossman School of Medicine, New York, NY USA

**Keywords:** Tau, Collagen-induced arthritis, Macrophage polarization, Inflammation, Autoimmune diseases

## Abstract

**Background:**

Tau protein serves a pro-inflammatory function in neuroinflammation. However, the role of tau in other inflammatory disorders such as rheumatoid arthritis (RA) is less explored. This study is to investigate the role of endogenous tau and the potential mechanisms in the pathogenesis of inflammatory arthritis.

**Methods:**

We established collagen-induced arthritis (CIA) model in wild-type and Tau-/- mice to compare the clinical score and arthritis incidence. Micro-CT analysis was used to evaluate bone erosion of ankle joints. Histological analysis was performed to assess inflammatory cell infiltration, cartilage damage, and osteoclast activity in the ankle joints. Serum levels of pro-inflammatory cytokines were measured by ELISA. The expression levels of macrophage markers were determined by immunohistochemistry staining and quantitative real-time PCR.

**Results:**

Tau expression was upregulated in joints under inflammatory condition. Tau deletion in mice exhibited milder inflammation and protected against the progression of CIA, evidenced by reduced serum levels of pro-inflammatory cytokines and attenuated bone loss, inflammatory cell infiltration, cartilage damage, and osteoclast activity in the ankle joints. Furthermore, tau deficiency led to the inhibition of classically activated type 1 (M1) macrophage polarization in the synovium.

**Conclusion:**

Tau is a previously unrecognized critical regulator in the pathogenesis of RA and may provide a potential therapeutic target for autoimmune and inflammatory joint diseases.

## Introduction

Rheumatoid arthritis (RA) is one of the most autoimmune and inflammatory disorders affecting approximately 2% world population, characterized by joint swelling and destruction accompanied by bone erosion and cartilage damage due to synovial inflammation [1]. A range of infiltrating immune cells are implicated in the pathogenesis of RA, mainly comprising macrophages, T cells, B cells, and mast cells [2, 3]. Among them, the macrophage is regarded as the core of mediating inflammation in the development of RA because of its two main distinct phenotypes: classically activated type 1 (M1) and alternatively activated type 2 (M2) macrophages [4, 5]. Distinct phenotypic macrophages play opposing roles in inflammatory arthritis [6]. M1 macrophages produce pro-inflammatory cytokines such as tumor necrosis factor α (TNF-α), interleukin-1β (IL-1β), and IL-6, which could activate fibroblasts to further generate pro-inflammatory factors, resulting in the acceleration of inflammation process [7]. Conversely, M2 macrophages are involved in the resolution of inflammation by the release of anti-inflammatory cytokines such as IL-10 [8].

Tau is defined as a microtubule-associated protein that functions in the regulation of microtubule assembly and stabilization [9, 10]. Compelling evidence have established the physiological and pathological function of tau in diverse neurodegenerative disorders, including Alzheimer’s disease (AD), frontotemporal dementia (FTD), Huntington disease (HD) [11–13]. Increasing evidence indicates that tau is implicated in various biological processes inside and outside of neurons by mediating multiple signaling pathways due to its diverse post-translational modifications [14, 15]. Emerging evidence from recent experimental studies indicate that tau plays a significant role in activating microglia and the aggregation of tau can directly induce microglia towards M1 phenotypes [16–18], manifesting that tau plays a pro-inflammatory role in neuroinflammation [19]. A significant finding was that tau-transgenic mice exhibited a greater susceptibility to collagen-induced arthritis (CIA), with an earlier onset and higher incidence than the control group [20]. This evidence suggests that tau may exert pro-inflammatory activity in other autoimmune and inflammatory diseases beyond neuroinflammation.

In the current study, we established CIA, an experimental model of human rheumatoid arthritis (RA) [21], in WT and Tau-/- mice to characterize the role of endogenous tau in inflammatory arthritis as well as the potential mechanism involved. Our findings demonstrate that tau deficiency could attenuate the progression of CIA by inhibiting M1 macrophage polarization, and targeting tau may provide a potential therapeutic strategy for treating autoimmune inflammatory joint diseases.

## Materials and methods

### Mice

C57BL/6 (000664) and Tau-/- (007251) mice were purchased form The Jackson Laboratory (Bar Harbor, ME, USA). Tau-/- mice were on the C57BL/6 background. All mice were housed on a 12-h light/dark cycle with free access to food and water in a specific pathogen‑free facility. All animal protocols were approved by the Institutional Animal Care and Use Committee of New York University.

### CIA induction and assessment

Twelve-week-old and weight-matched WT and Tau-/- male mice were randomly divided into two groups: the control group and the CIA group, respectively. CIA group mice were immunized intradermally with 100μL emulsion containing an equal volume of chicken type II collagen (20,012, Chondrex) and complete Freund’s adjuvant (7001, Chondrex) at the base of tail (day 0) [22–24]. Booster injections were performed with chicken type II collagen emulsified in incomplete Freund’s adjuvant (7002, Chondrex) on day 19 [25, 26]. Due to the mice on the C57BL/6 background having up to 60% arthritis incidence, the mice with CIA phenotype were included for further study. The mice without CIA phenotype were excluded from the study but could be used for the calculation of arthritis incidence. There were at least 6 control mice or 6 mice with CIA phenotype in the control group or the CIA group, respectively. After the booster injection, the clinical score was assessed on each paw every other day for five weeks using the following system: 0 = no erythema and swelling; 1 = erythema and mild swelling confined to the tarsals or ankle joint; 2 = erythema and mild swelling extending from the ankle to the tarsals; 3 = erythema and moderate swelling extending from the ankle to metatarsal joints; 4 = erythema and severe swelling encompass the ankle, foot and digits, or ankylosis of the limb [27]. Each paw was scored and scores of four paws were summed to give a total clinical score with a maximum possible score of 16 for each mouse. Arthritis incidence was defined as the ratio of the number of mice with CIA phenotype to a total number of immunized mice, multiplied by 100. After Five weeks after boost injections, the mice were sacrificed (day 55), hind paws and sera were harvested for further study.

### Micro-CT analysis

Hind paws from WT and Tau-/- mice were fixed in 4% paraformaldehyde (PFA) for 24 h and then stored in 70% ethanol. After fixation, hand paws were scanned using a Scanco vivaCT40 cone-beam scanner (SCANCO Medical, Switzerland) with a source voltage of 55 kV, a source current of 145 μA, and a resolution of 10.5 μm. After reconstruction, the parameters of trabecular bone of distal tibia, including trabecular bone mineral density (Tb.BMD, g/cm^3^), bone volume/tissue volume (BV/TV, %), trabecular thickness (Tb.Th μm), and trabecular number (Tb.N, 1/mm), were analyzed using CT Analyser (CTan) v.1.18.8.0 (Bruker). The micro-CT images were obtained by CTvox v.3.3.1 software (Bruker).

### Histological analysis

Hind paws were decalcified with 10% EDTA for four weeks and embedded in paraffin. Serial paraffin Sects. (5 μm) were prepared and stained with hematoxylin and eosin (H&E) to evaluate the inflammation score and bone destruction score. Inflammation scores were obtained based on the following criteria: 0 = no inflammation; 1 = slight thickening of the lining layer or some infiltrating cells in the underlying layer; 2 = slight thickening of the lining layer plus some infiltrating cells in the underlying layer; 3 = thickening of the lining layer, an influx of cells in the underlying layer, and presence of cells in the synovial space; 4 = synovium highly infiltrated with many inflammatory cells [6]. Safranin O staining was performed for cartilage damage assessment, which was based on the following score system: 0 = normal; 1 = minimal erosion limited to single spots; 2 = slight to moderate erosion in a limited area; 3 = more extensive erosions; 4 = general destruction [28]. Sections were stained for tartrate-resistant acid phosphatase (TRAP) to identify the osteoclasts. All images were captured using the Zeiss microscope (Axio Scope A.1, Carl Zeiss, LLC). The quantification of osteoclasts was performed with ImageJ software.

### Immunohistochemistry staining

Paraffin sections were deparaffinized and rehydrated through xylenes and graded ethanol series. Then sections were pre-treated with 0.1% trypsin for antigen retrieval (30 min, 37 °C), followed by incubation with 3% H_2_O_2_ (30 min, 4 °C). After incubation with blocking buffer (3% BSA + 20% goat serum) for 60 min at 37 °C, sections were treated with primary antibodies against tau (1:250, PA5-29,610, Invitrogen), iNos (1:100, ab15323, Abcam), CD206 (1:8000, ab64693, Abcam) and F4/80 (1:100, ab6640, Abcam) overnight at 4 °C. The next day, the sections were incubated with biotinylated secondary antibodies for 60 min at room temperature. After washing with PBS, the Vectastain Elite ABC kit (PK-6100, Vector) was used to amplify signals. Sections were then incubated with 0.5 mg/ml 3,3-diaminobenzidine (DAB) in 50 mM Tris–Cl substrate (Sigma-Aldrich) until desired stain intensity developed. After this, sections were counterstained with 1% methyl green, cleared, and mounted. All images were obtained using the Zeiss microscope. The quantification analysis was performed with ImageJ software.

### Isolation of primary bone marrow derived macrophages (BMDMs) and M1 and M2 polarization

Bone marrow cells were extracted from twelve-week-old WT and Tau-/- mice and cultured in αMEM supplemented with 10% FBS plus M-CSF (10 ng/mL, Biolegend) for 5 days. After bone marrow cells were differentiated into macrophages, the cells were treated with LPS (100 ng/mL, Sigma-Aldrich) plus IFN-γ (20 ng/mL, Peprotech) or IL-4 (20 ng/mL, Peprotech) for 18 h to polarize cells to M1 or M2 macrophages, respectively.

### Quantitative real-time PCR

Total RNA was extracted from cells using TRIzol reagent according to the manufacturer’s instructions. cDNA was prepared using 1 μg RNA with SuperScript® Reverse Transcriptase (M314C, Promega Corporation). Quantitative real-time PCR (qRT-PCR) was performed using SYBR® Green PCR Master Mix (4,309,155, Applied Biosystems). The prepared reaction was conducted using StepOnePlusTM real-time PCR Systems (Applied Biosystems). The following primers were used: *Tau* (forward: CCTGAGCAAAGTGACCTCCAAG; reverse: CAAGGAGCCAATCTTCGACTGG); *IL-6* (forward: TTCCATCCAGTTGCCTTCTTG; reverse: AGGTCTGTTGGGAGTGGTATC); *Nos2* (forward: TGTTAGAGACACTTCTGAGGCTC; reverse: ACTTTGGATGGATTTGACTTTGAAG); *Arg1* (forward: TGCCAAAGACATCGT GTACATTG; reverse: CTTCCCAGCAGGTAGCTGAAG); *Mgl1* (forward: CAGATCCGTATCTGTCTGGATC; reverse: AGGTGGGTCCAAGAGAGGATG). Target gene mRNA levels were analyzed using ΔΔCT method. Relative mRNA fold changes were normalized to *Gapdh*.

### Western blot

Raw264.7 cells were treated with PBS or TNF-α (30 ng/mL, PHC3015, Invitrogen) for 48 h and total protein was then extracted. The protein samples were separated using SDS-PAGE and then transferred a nitrocellulose membrane (1,620,097, Bio-Rad) using a wet transfer system. 5% (w/v) non-fat milk in TBST was used to block the membrane for 1 h at room temperature. The membrane was incubated with anti-Tau antibody (1:1000, MA5-12,808, Invitrogen) or GAPDH (1:1000, 60,004-I-Ig, Proteintech) at 4 °C overnight. After three times washing, horseradish peroxidase conjugated anti-mouse IgG (1:10,000, 115–035-003, Jackson ImmunoResearch Laboratories) was added for 1 h at room temperature. After incubated with chemiluminescent (ECL) substrate (NEL104001, Thermo Fisher Scientific), the bands on the membrane were detected using ChemiDoc Imaging System (17,001,401, Bio-Rad). The quantification of bands was performed using ImageJ software.

### ELISA

The concentrations of IL-1β and IL-6 were measured in serum collected from mouse models using ELISA kits, according to product specifications (IL-1β: 88–7013-88, Invitrogen; IL-6: 88–7064-88, Invitrogen).

### Statistical analysis

Statistical analysis was performed using GraphPad Prism 8 statistic software (GraphPad). All data were presented as mean ± standard errors (SD). Statistical significance between two groups was analyzed using two-tailed, unpaired Student’s *t*-tests. Comparison of multiple groups was analyzed using two-way ANOVA. A value of *P* was considered statistically significant if *P* < 0.05.

## Results

### The expression of tau is increased in macrophages and synovium of joints under inflammation conditions

**Fig. 1:**
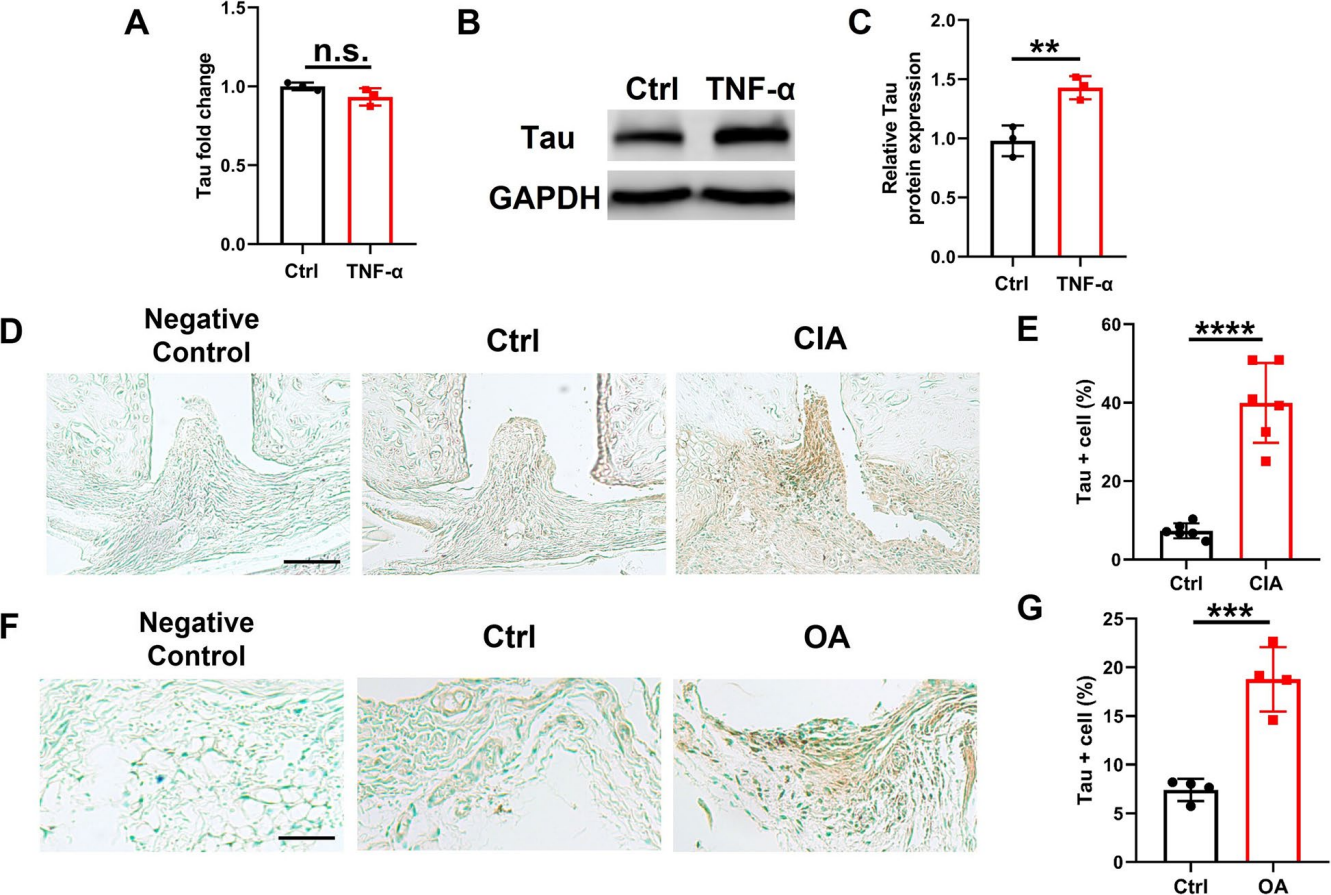
Tau protein level is increased in inflammation conditions. **A** qRT-PCR analysis of *Tau* mRNA level in Raw264.7 cells treated with PBS or TNF-α (30 ng/mL) for 24 h. **B** Western blot analysis of tau protein in Raw264.7 cells treated with PBS or TNF-α (30 ng/mL) for 48 h. **C** Densitometry analysis of Western blot results shown in (**B**). **D** Representative images of immunohistochemistry staining of tau in the synovium of ankle joints from WT mice with or without CIA at 7 weeks after immunization. Scale bar: 100 µm. **E** Quantification of tau positive cells in the synovium of ankle joints. *n* = 6 mice for each group. **F** Representative images of immunohistochemistry staining of tau in the synovium of knee joints from sham or DMM operated WT mice at 12 weeks after surgery. Scale bar: 50 µm. **G** Quantification of tau positive cells in the synovium of knee joints. *n* = 4 mice for each group. **p < 0.01; ***p < 0.001; ****p < 0.0001. CIA, collagen-induced arthritis; OA, osteoarthritis; DMM, destabilization of the medial meniscus

To examine whether tau expression altered under inflammatory conditions, we first measured tau mRNA and protein levels in Raw264.7 cells after TNF-α stimulation. Tau protein level was significantly elevated following TNF-α stimulation while mRNA level remained unchanged (Fig. 1A-C), suggesting inflammation could regulate tau expression at posttranscriptional level. To further determine the alteration of tau expression level in vivo, we performed immunohistochemical staining on ankle joint sections from wild-type (WT) mice with or without CIA. The results revealed that tau expression was dramatically increased in the synovial tissues of CIA mice compared with those of the control group (Fig. 1D, E). Besides, osteoarthritis (OA) is also considered to be a mild chronic inflammatory disease [29], we also determined whether tau expression changed in the synovium of surgically induced destabilization of medial meniscus OA mice. In line with the results obtained from CIA mice, tau expression was also enhanced in the synovium of knee joints from OA mice compared with that from the control group (Fig. 1F, G). Taken together, the expression of tau is increased in macrophages and synovium of joints under inflammation conditions.

### Tau deficiency reduces arthritis severity

**Fig. 2:**
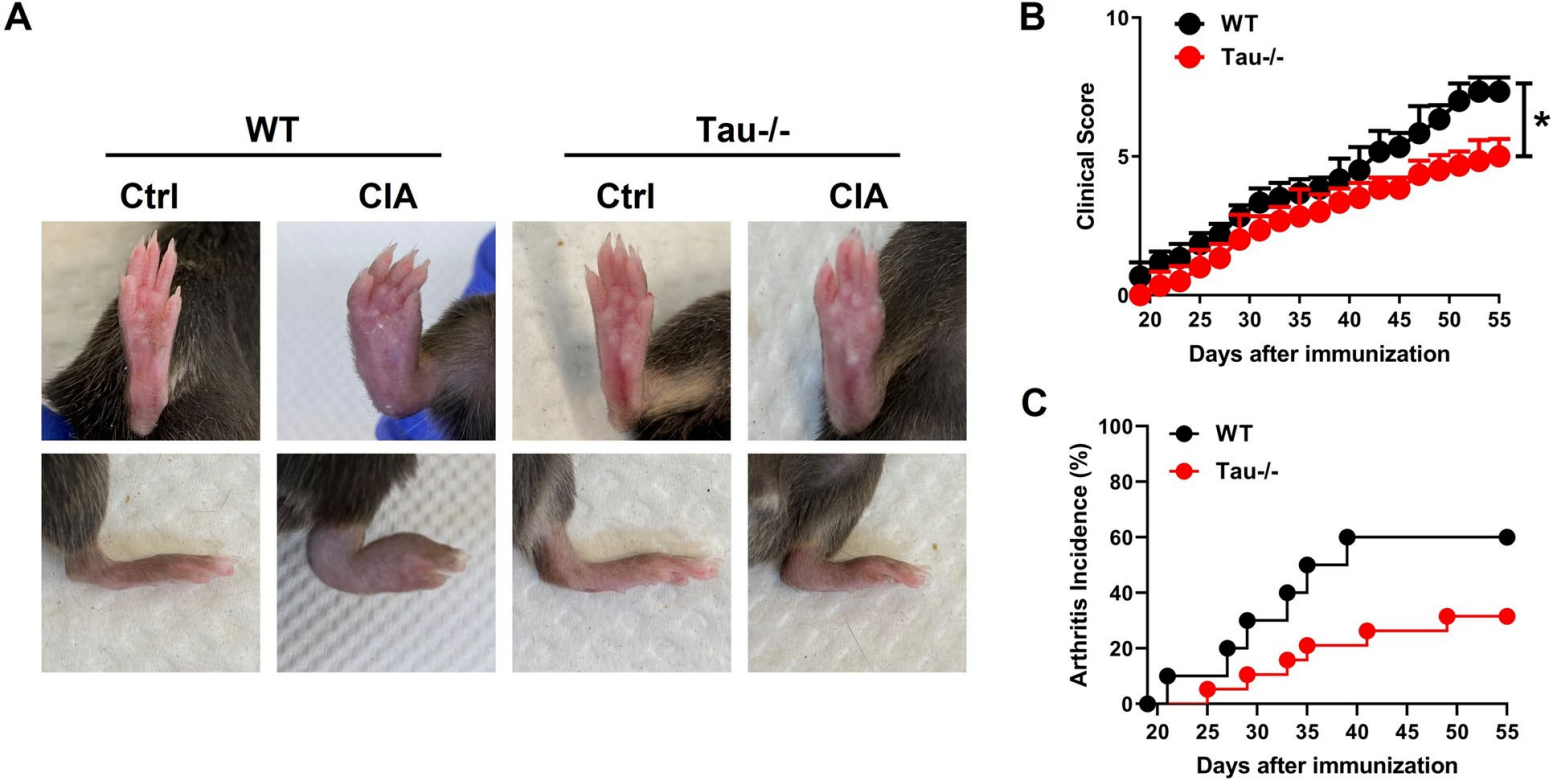
Tau deficiency attenuates the onset and progression of CIA. **A** Representative images of hind paws of WT and Tau-/- mice with CIA. **B**, **C** Clinical score (**B**) and arthritis incidence (**C**) of WT and Tau-/- mice with CIA model. *n* = 6 mice for each group. **p < 0.01

To determine the role of endogenous tau in the pathogenesis of RA, we established CIA model in WT and Tau-/- mice [30, 31]. The absence of tau led to a reduction in hind paw swelling compared to WT mice following CIA induction (Fig. 2A). Accordingly, tau deletion reduced clinical score, delayed the onset of disease, and decreased disease incidence (Fig. 2B, C).

**Fig. 3:**
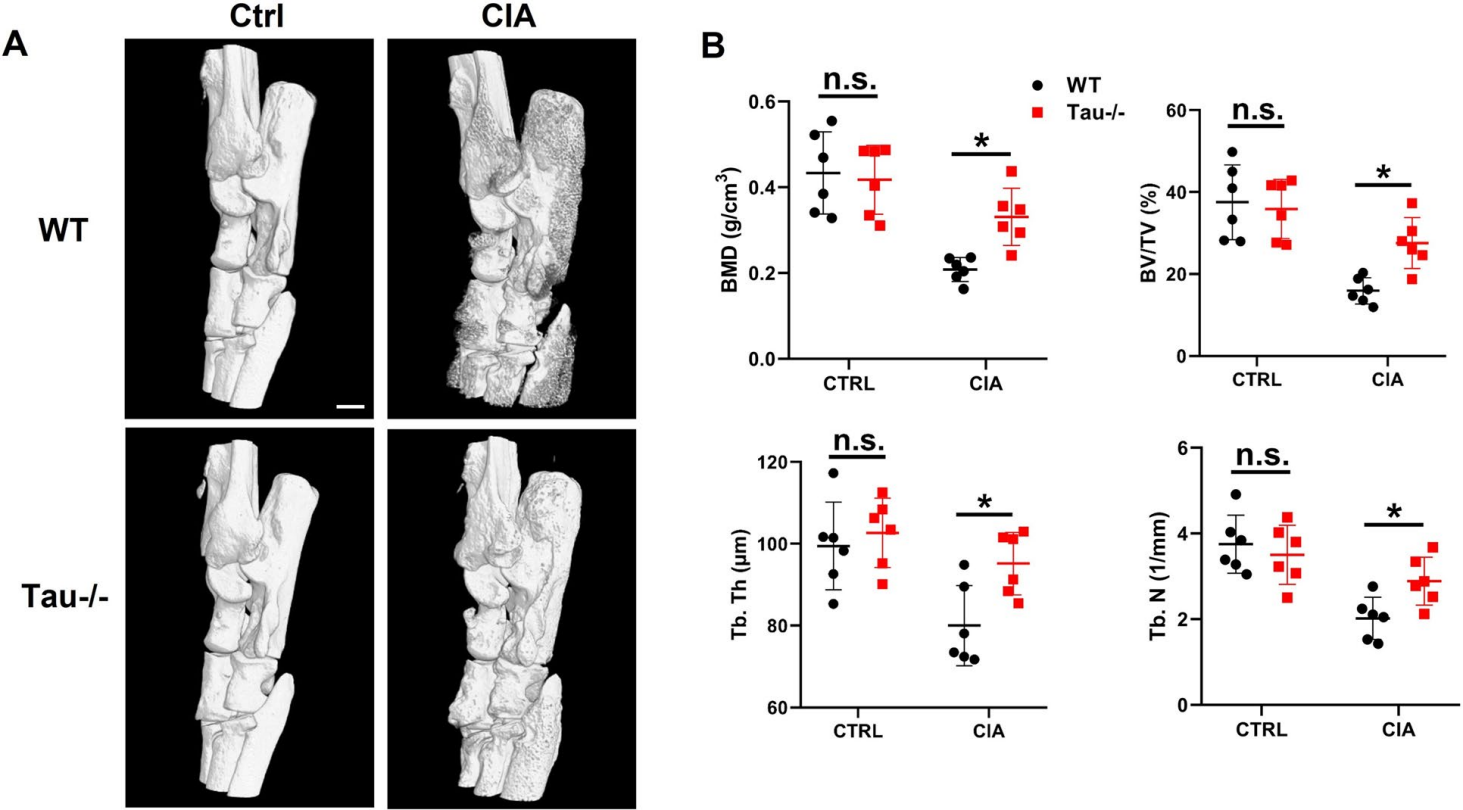
Ankle joint destruction is alleviated in Tau-/- mice with CIA. **A** Representative micro-CT images of hind paws of WT and Tau-/- mice. Scale bar: 500 µm. **B** Bone mineral density (BMD), bone volume/tissue volume (BV/TV), trabecular thickness (Tb. Th), and trabecular number (Tb. N) in the distal tibia were analyzed by micro-CT. *n* = 6 mice for each group. *p < 0.05

To further investigate the effect of tau deletion on arthritis associated bone erosion, we performed Micro-CT analysis on ankle joints from WT and Tau-/- mice on day 55 of CIA. Micro-CT images demonstrated that ankle joints of WT mice exhibited considerable bone erosion associated with arthritis, while ankle joints of Tau-/- mice showed significantly less bone erosion (Fig. 3A). The reduced bone erosion in Tau-/- CIA mice was also quantitatively evidenced by the increase in bone mineral density (BMD), bone volume/tissue volume (BV/TV), trabecular thickness (Tb. Th), and trabecular number (Tb. N) in the distal tibias compared to those in WT CIA mice (Fig. 3B). Collectively, these findings clearly demonstrate that tau deficiency in mice leads to decreased susceptibility to CIA.

### Tau deletion alleviates inflammation and bone loss

**Fig. 4:**
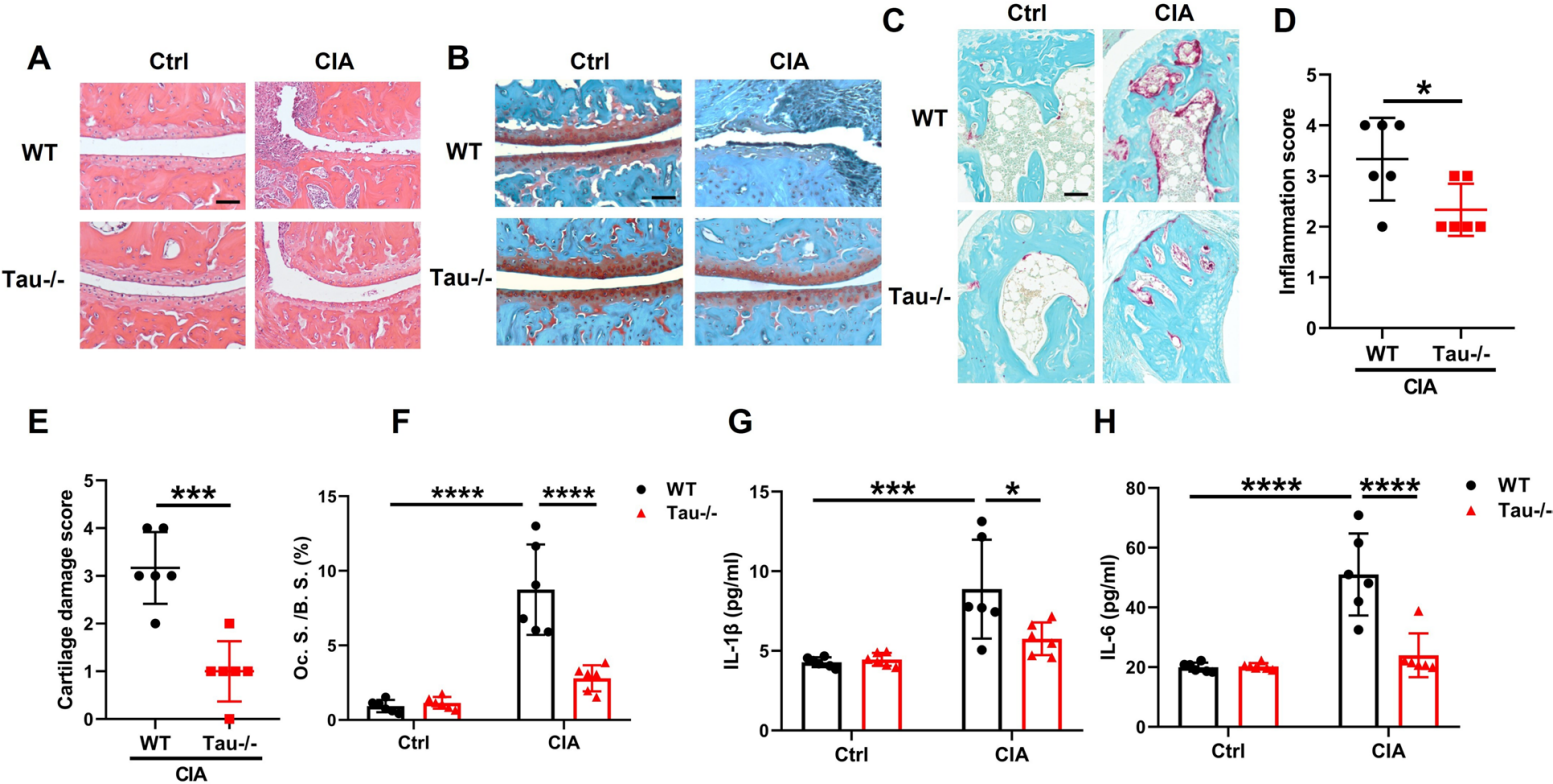
Tau deletion decreases the inflammation of ankle joints in CIA mice. **A**-**C** Representative images of H&E (**A**), Safranin O (**B**), and TRAP staining (**C**) in ankle joints from WT and Tau-/- mice with or without CIA. Scale bar: 100 µm. **D**, **E** Inflammation score (**D**) and cartilage damage score (**E**) were evaluated according to the scoring system. **F** Histomorphometric analysis of osteoclast surface per bone surface (Oc.S/BS). **G**, **H** Serum levels of IL-1β (**G**) and IL-6 (**H**) were measured by ELISA. *n* = 6 mice for each group. *p < 0.05; **p < 0.01; ***p < 0.001; ****p < 0.0001

Enhanced inflammatory cell infiltration, cartilage damage, and osteoclast activity in the ankle joints are typical hallmarks during the progression of CIA [32, 33]. Histopathological and quantitative analysis on the H&E stained ankle joints showed that WT CIA mice have abundant inflammatory cell infiltration, whereas Tau-/- CIA mice exhibited a significant reduction of inflammatory cell inflammation (Fig. 4A, D). Safranin O staining demonstrated that Tau-/- mice were markedly protected from arthritis-associated cartilage loss observed in WT mice (Fig. 4B, E). In addition, tartrate-resistant acid phosphatase (TRAP) staining revealed abundant osteoclasts in WT CIA mice, and tau deletion could markedly protect against arthritis-associated osteoclasts formation and bone destruction (Fig. 4C, F). Accordingly, the serum levels of pro-inflammatory cytokines, such as IL-1β and IL-6, significantly declined in Tau-/- mice with CIA (Fig. 4G, H). The results demonstrate that Tau-/- mice have milder inflammatory arthritis phenotypes and tau deletion significantly protects against the development of inflammatory arthritis.

### Loss of tau inhibits M1 macrophage polarization

**Fig. 5:**
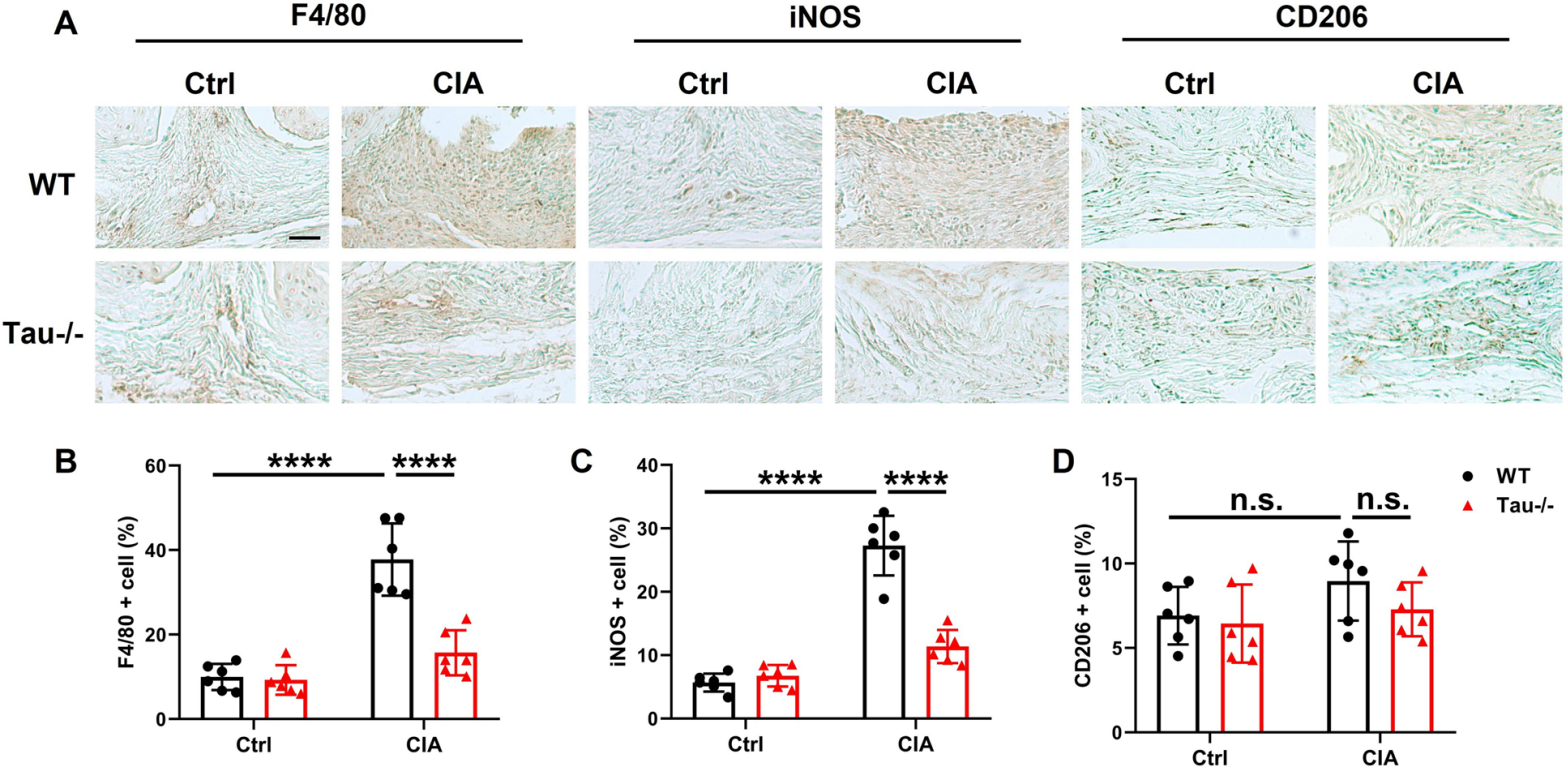
Tau deletion inhibits macrophage infiltration and M1 polarization in CIA. **A** Representative images of immunohistochemical staining of F4/80, iNOS and CD206 in the synovium of ankle joints from WT and Tau-/- mice with or without CIA. Scale bar: 100 µm. **B**-**D** Quantification of F4/80 positive (**B**), iNos positive (**C**) and CD206 positive (**D**) macrophages in the synovium of ankle joints from WT and Tau-/- mice with or without CIA. *n* = 6 mice for each group. ****p < 0.0001

To understand the mechanisms underlying the protective effects of tau deficiency against inflammatory arthritis, we sought to determine whether tau deletion could have an impact on synovial macrophages infiltration and polarization, which are the most abundant cells in the synovium and strongly associated with the pathogenesis of RA [34, 35]. The immunohistochemistry staining for F4/80, a marker of general macrophage, revealed that the total number of macrophages in the synovium of ankle joints from WT mice with CIA was much higher than that of Tau-/- CIA mice (Fig. 5A, B). We next explored the effects of tau deletion on macrophage polarization. The results suggested that the expression of iNOS, a marker for M1 macrophage, in Tau-/- CIA mice was obviously lower than that in WT CIA group (Fig. 5A, C). However, tau deletion appeared to have no effect on M2 macrophage, as evidenced by unchanged number of CD206 positive cells between WT and Tau-/- mice with CIA (Fig. 5A, D). Notably, the deletion of tau did not alter the macrophage infiltration and macrophage polarization under steady condition **(**Fig. 5A-D).

**Fig. 6:**
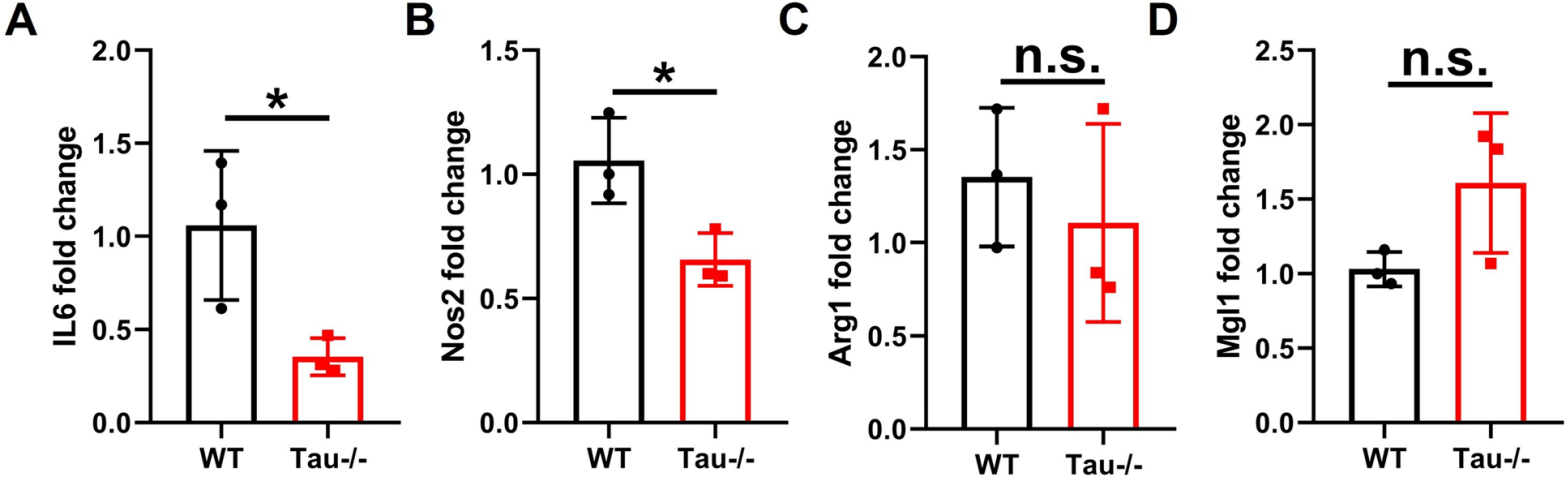
Tau deletion inhibits macrophage skew towards M1 in vitro. **A**, **B** mRNA levels of *Il6* (**A**) and *Nos2* (**B**) in WT and Tau-/- BMDMs polarized to M1. **C**, **D** mRNA levels of *Arg1* (**C**) and *Mgl1* (**D**) in WT and Tau-/- BMDMs polarized to M2. *n* = 3 biological replicates. *p < 0.05

To examine the role of tau in macrophage polarization in vitro, we isolated bone marrow–derived macrophages (BMDMs) from WT and Tau-/- mice, which were then polarized to M1 and M2 macrophages, respectively [6, 24]. As shown in Fig. 6A and B, tau deletion significantly reduced the expressions of M1-assocaited genes *Il6* and *Nos2*, without affecting the expressions of M2-associated genes *Arg1* and *Mgl1* (Fig. 6A-D). In sum, the findings in this study indicate that tau deletion suppresses inflammation by inhibiting M1 macrophage polarization both in vitro and in vivo.

## Discussion

Tau is well recognized as a microtubule-related protein, abundantly found in brain cells (neurons), and closely associated with the pathogenesis of various neurodegenerative diseases [15, 36]. Numerous studies regarding the function of tau protein mainly focus on neuroinflammation [37–39]. However, the role of tau in autoimmune and inflammatory diseases remains largely unknown. In the current study, we demonstrated a previously unrecognized role of tau in CIA, a common model for human RA, by controlling macrophage infiltration and polarization. We observed that tau protein expression level was increased after inflammation stimulation in macrophage in vitro. Accordingly, its expression level was up-regulated in the synovium under inflammatory conditions in both OA and CIA. We further revealed that tau deletion mice were less sensitive to CIA. These results manifest that tau plays a critical role in inflammatory arthritis and suggest manipulation of tau expression and function could be a promising therapeutic target for autoimmune and inflammatory disorders.

RA, caused by autoimmune dysfunction, is defined as a chronic and inflammatory joint disease, characterized by infiltration of the synovial membrane with diverse immune cells as well as neovascularization, progressively leading to the destruction of articular cartilage and bone [40–42]. Besides, another prominent feature of RA is the substantial enhancement of osteoclast activity due to inflammatory responses, leading to intense bone loss [43], also known as inflammation-induced bone loss. There are three forms of bone loss in RA: (1) focal articular bone erosion; (2) periarticular osteopenia adjacent to inflamed joints; (3) generalized osteoporosis [44, 45]. The treatment strategies in RA have evolved dramatically in the past two decades, aiming at prevention of the onset of RA, inhibition of inflammation, prevention of disability to maintain normal quality of life [46, 47]. Here, tau deletion exhibited anti-inflammation effect in the course of CIA, including delayed disease onset, downregulation of the serum pro-inflammatory cytokines IL-1β and IL-6, and attenuated inflammatory cell infiltration, cartilage damage and bone erosion in the ankle joints. In particular, decreased number of osteoclasts caused by tau deletion contributed to milder bone loss in ankle joints of CIA mice compared with WT groups. These findings imply that tau is involved in the pathological process of RA and tau deficiency could attenuate the inflammation and bone loss during the progression of RA.

Macrophages, belonging to myeloid immune cells, play an essential role in coordinating the inflammatory process of RA by interacting with other immune cells, including T cells, B cells, neutrophils, synovial fibroblasts, and so on [48–50]. Macrophages and functionally heterogeneity and extracellular cues regulate the polarization of macrophages, which comprise two distinguishing phenotypes: classically activated macrophages (M1) and alternatively activated macrophages (M2) [51, 52]. M1 polarization can be induced by some pro-inflammatory stimuli, such as granulocyte–macrophage colony stimulating factor (GM-CSF) and lipopolysaccharide (LPS) [53]. M1 macrophages are considered as pro-inflammatory cells due to the high production of pro-inflammatory cytokines through the activation of nuclear factor kappa B (NF-κB) signaling pathway, including TNF-α, IL-1β, and IL-6 [54], which play an important role in aggravating the inflammatory response. Nevertheless, M2 macrophages serve as anti-inflammatory cells and are generally activated by macrophage colony stimulating factor (M-CSF) and IL-4 [55]. M2 macrophages are responsible for shifting inflammation activation toward inflammation inhibition by high expression levels of IL-10 and transforming growth factor-β (TGF-β), therefore alleviating inflammatory responses [56, 57]. Inducible nitric oxide synthase (iNOS) and cluster of differentiation 206 (CD206), highly expressed in M1 and M2 macrophages, are thought as specific markers for M1 and M2 phenotypes, respectively [50]. Increasing evidence demonstrates that the balance between M1 and M2 is particularly important for the pathogenesis of RA. In the current study, we revealed that tau deletion inhibited M1 macrophage polarization in vitro and in vivo, which is in line with the previous studies that tau induces microglia into M1 phenotype in neurodegenerative disorders [17, 58, 59]. Thus, tau exerts pro-inflammatory effects via skewing macrophages towards M1 macrophages in autoimmune and inflammatory joint disorders. In the current study, we mainly focus on the role of tau protein in M1 polarization and its impact on CIA. One limitation of this study is that the mechanism behind the effect of tau deficiency has not been fully investigated. The exploration of accurate mechanism of M1 macrophage polarization inhibition caused by tau deficiency will be addressed in our future research endeavors.

## Conclusions

Taken together, these findings unveil the important function of tau in the inflammatory arthritis and indicate that tau deficiency could reduce inflammatory reactions by inhibiting the polarization of macrophages into M1 phenotype, thereby preventing bone loss and protecting against the progression of CIA. Our work offers the evidence for better understanding the pathogenesis of RA and provides a potential therapeutic strategy for autoimmune and inflammatory diseases.

## Data Availability

All data needed to evaluate the conclusions in the paper are present in the paper. Additional data related to this paper may be requested from the authors.

## Abbreviations

RA: Rheumatoid arthritis
CIA: Collagen-induced arthritis
M1: Type 1 macrophages
M2: Type 2 macrophages
TNF-α: Tumor necrosis factor α
IL-1β: Interleukin-1β
AD: Alzheimer’s disease
FTD: Frontotemporal dementia
HD: Huntington disease
Tb.BMD: Trabecular bone mineral density
BV/TV: Bone volume/tissue volume
Tb.Th: Trabecular thickness
Tb.N: Trabecular number
TRAP: Tartrate-resistant acid phosphatase
BMDMs: Bone marrow derived macrophages
qRT-PCR: Quantitative real-time PCR
WT: Wild-type
OA: Osteoarthritis
iNOS: Inducible nitric oxide synthase
CD206: Cluster of differentiation 206
